# Aggregation of Sea Urchin Phagocytes Is Augmented *In Vitro* by Lipopolysaccharide

**DOI:** 10.1371/journal.pone.0061419

**Published:** 2013-04-17

**Authors:** Audrey J. Majeske, Christopher J. Bayne, L. Courtney Smith

**Affiliations:** 1 Department of Biological Sciences, George Washington University, Washington, D. C., United States of America; 2 Department of Zoology, Oregon State University, Corvallis, Oregon, United States of America; Chang Gung University, Taiwan

## Abstract

Development of protocols and media for culturing immune cells from marine invertebrates has not kept pace with advancements in mammalian immune cell culture, the latter having been driven by the need to understand the causes of and develop therapies for human and animal diseases. However, expansion of the aquaculture industry and the diseases that threaten these systems creates the need to develop cell and tissue culture methods for marine invertebrates. Such methods will enable us to better understand the causes of disease outbreaks and to develop means to avoid and remedy epidemics. We report a method for the short-term culture of phagocytes from the purple sea urchin, *Strongylocentrotus purpuratus*, by modifying an approach previously used to culture cells from another sea urchin species. The viability of cultured phagocytes from the purple sea urchin decreases from 91.6% to 57% over six days and phagocyte morphology changes from single cells to aggregates leading to the formation of syncytia-like structures. This process is accelerated in the presence of lipopolysaccharide suggesting that phagocytes are capable of detecting this molecular pattern in culture conditions. Sea urchin immune response proteins, called Sp185/333, are expressed on the surface of a subset of phagocytes and have been associated with syncytia-like structures. We evaluated their expression in cultured phagocytes to determine their possible role in cell aggregation and in the formation of syncytia-like structures. Between 0 and 3 hr, syncytia-like structures were observed in cultures when only ∼10% of the cells were positive for Sp185/333 proteins. At 24 hr, ∼90% of the nuclei were Sp185/333-positive when all of the phagocytes had aggregated into syncytia-like structures. Consequently, we conclude that the Sp185/333 proteins do not have a major role in initiating the aggregation of cultured phagocytes, however the Sp185/333 proteins are associated with the clustered nuclei within the syncytia-like structures.

## Introduction

The advancement of methods for culturing mammalian immune cells has proceeded ahead of progress for developing cell culture methods for lower vertebrates and invertebrates. However, with the expansion of the aquaculture industry, disease outbreaks are a major cause of death among aquacultured species due to infections by bacteria, fungi, viruses and parasites [1]. Therefore, the development of methods for *in vitro* culture of cells from marine invertebrates has been driven by the need to understand these epidemics and to develop a means to investigate and remedy future outbreaks. Cell and tissue culture systems also provide tools to study basic biological functions of marine organisms including the evaluation of responses to toxins and pathogens. Furthermore, cell culture systems have some benefits over experiments using whole animals. Several marine invertebrates have been subjects of cell culture studies, including species of shrimp, crabs, crayfish, molluscs, ascidians, sea stars, sea cucumbers and sea urchins (reviewed in [2]). The subject of this study, the California purple sea urchin, *Strongylocentrotus purpuratus*, is important not only to aquaculture (mainly for harvesting ovaries [Japanese *uni*] for human consumption), but these animals have been used in studies of developmental biology for over 100 years (reviewed in [3]). Because sea urchins and other classes of echinoderms are close relatives to the chordates, these species provide clues to important evolutionary processes within the deuterostome lineage of animals that includes the vertebrates [4].

*In vitro* culture of sea urchin coelomocytes (immune cells) was first undertaken between the late 1960’s and the early 1980’s [5]–[11]. Methods for the short- and/or long-term culture of immune cells from marine invertebrates were developed to describe the morphology and activities of these cells [5]–[9], [12]–[15]. Some of the long-term goals of cell culture for aquacultured species have been to 1) meet the commercial demand for sources of new biologically active chemical compounds with pharmaceutical activities, and 2) provide scientific tools to study endocrinology and pathology of edible species [16]. However, marine invertebrate cell culture is still in its infancy with respect to meeting these long-term goals. Although previous work has reported culture methods to evaluate the biological functions of marine invertebrate cells, in many cases a basic evaluation of the methods are lacking. For example, reports state that immune cells from marine invertebrates in culture remain viable throughout the duration of the experiments, however, only one [15] of nine studies [5]–[9], [12]–[15] reported results for cell viability. There are also multiple reports that immune cells aggregate into syncytia in culture over time [5], [8], [9], [11], [13]. It has long been assumed that these aggregates are true syncytia, i.e., that the plasma membranes between the neighboring cells have fused forming giant multinucleated structures. However, there are no experimental data other than simple observation to support this notion. Because of this, we refer to the aggregated cells in this study as syncytia-like structures.

The main components of successful culture media for sea urchin coelomocytes and other marine invertebrate immune cells include a nutrient-rich medium developed for mammalian cell culture, a buffering agent, and a mixture of salts to mimic the natural environment in which the animals live. Components used in culture media for coelomocytes include: Hepes-sea water medium (HSM) with Dulbecco’s Modified Eagle Medium (MEM) [5]–[9] and Jamarin U (commercially available filtered sea water from Jamarin Laboratory, Osaka, Japan) with fetal calf serum (FCS) [13]. Similar immune cell culture media for hemocytes or hemopoietic stem cells from crustaceans include Leibovitz’s L-15 Medium (L-15) [17] with FCS and artificial salt water [12], [14], [15]. The time span for which coelomocytes from several sea urchin species have been able to survive in culture ranges from a few days to three months [9], [11], [13] and hemocytes from crabs and shrimp have been cultured for two weeks to three months [12], [14], [15].

To initiate cell cultures, the peristomial membranes of adult sea urchins are punctured with hypodermic needles and coelomic fluid containing coelomocytes is withdrawn from the coelomic cavity. Three morphologically and (likely) functionally distinct types of coelomocyte are suspended in coelomic fluid, which includes vibratile cells, spherule cells (red and colorless), and phagocytes (of which there are three types; reviewed in [18], [19]). The phagocyte class includes polygonal cells, which display cytoskeletal morphology with actin cables oriented along the axes of the cells [20]–[22]. Discoidal cells are also large phagocytes that have cytoskeletal actin cables oriented radially giving them a fried egg appearance. Both polygonal and discoidal cells can readily change the morphology of their pseudopods from filopodial to lamellipodial and back. Small phagocytes are significantly smaller than polygonal and discoidal cells [23], and display a filopodial cytoskeleton that does not change to lamellipodial [24]. Phagocytes comprise the majority of the coelomocytes, and in short-term cultures have been shown to phagocytose yeast, variously treated and opsonized red blood cells, several types of beads, small particles such as colloidal gold and carbon, labeled molecules, and bacteria (reviewed in [18], [25], [26] and see citations within). Coelomocytes wall off clusters of bacteria in hanging drop cultures [10] and quickly clear injected bacteria from the coelomic cavity [7], [27], [28]. Upon removal from a sea urchin, coelomocytes immediately initiate clot formation indicative of immune effector cell activation (mainly phagocytes) near the site of the needle puncture wound from which the coelomocytes are collected [11], [29]. Moreover, coelomocytes are typically collected through a sterile needle, which most likely causes shear stresses on the cells and may promote clotting. In the intact animal, both protein and cellular clots may be responses to injury and likely prevent the loss of coelomic fluid through “bleeding.” Amassin and arylsulfatase function as mediators of clotting in sea urchins [30], [31]. Amassin becomes cross-linked by disulfide bonds forming a protein clot in which coelomocytes are captured or entangled. Arylsulfatase may be involved in crosslinking polysaccharides on cell surfaces forming cellular clots. Activation of phagocytes induces a morphological change from lamellipodia to filopodia, which become sticky, inter-twine, and form cellular clots [21], [32]. To block the swift clotting reactions, anticoagulant is typically mixed with the coelomic fluid during coelomocyte collection and before placing the cells in culture. Once in culture and after removal of the anticoagulant, immune cells from both echinoderms and crustaceans aggregate over time into large syncytia-like structures, which form on glass [5], [9] and plastic surfaces (AJM, personal observations), as well as in hanging drop cultures in response to foreign particles and bacteria [10], [11], [13].

Here we report a modified method for short-term primary cultures of phagocytes from the purple sea urchin that is based on previous coelomocyte culture methods [33]. We report cell viability for the short-term cultures and utilize our method to investigate the changing morphology of the phagocytes and their responses to lipopolysaccharide (LPS). We also evaluate the expression of the Sp185/333 immune response proteins that are produced by two subsets of phagocytes (reviewed in [34], [35]). The family of Sp185/333 proteins is highly diverse, with up to 260 isoforms characterized from an individual sea urchin [36]. The general protein structure has a relatively conserved hydrophobic N-terminal signal sequence, a glycine-rich region, an integrin binding motif, a histidine-rich region and a C-terminal domain [37], [38]. These proteins tend to multimerize irreversibly into complexes with larger molecular weights and broader isoelectric points than predicted from message sequences [24], [36], [38], [39]. The Sp185/333 proteins are expressed by the polygonal phagocytes and small phagocytes, and are associated with the membranes of perinuclear vesicles as well as on the cell surface of the small phagocytes [24], [40]. Following immune challenge, the proportion of Sp185/333-positive (Sp185/333^+^) phagocytes in the coelomic fluid of adult sea urchins increases significantly [24], [41], however, it is not known whether the expression of Sp185/333 proteins can be induced in cultured phagocytes in response to immune challenge. Syncytia-like structures of phagocytes that form in very short-term cultures (<1 hr) show a striking association with Sp185/333 proteins [18], [24]. This has led to speculation that the combination of Sp185/333 protein multimerization plus their expression on the small phagocytes may initiate cell-cell associations that lead to aggregation and the formation of syncytia-like structures in culture. To address these questions, short-term, primary cultures of coelomocytes were established using cells collected from adult sea urchins. After settling in culture, the adherent phagocytes were the only cell types that remained attached to the culture well after media exchange. Consequently, our analyses were an evaluation of the polygonal, discoidal, and small phagocytes. Cell viability decreased to 57.0% over 6 days, and syncytia-like structures were evident in 3 hr cultures and beyond. The rate of phagocyte aggregation increased in response to LPS, however, phagocyte aggregation occurred in the absence of elevated Sp185/333 protein expression, suggesting that the Sp185/333 proteins are not required for cell aggregation.

## Materials and Methods

### Sea Urchins

Purple sea urchins, *Strongylocentrotus purpuratus*, were purchased from either Marinus Scientific Inc. (Long Beach, CA) or the Southern California Sea Urchin Co. (Corona del Mar, CA). Sea urchins were maintained as described [23] and fed weekly with commercial rehydrated kelp (Quickspice, Inc., Commerce, CA).

Some animals (*n* = 3) chosen for the study were immunoquiescent (Iq) resulting from long-term housing in the laboratory for more than eight months in a saltwater aquarium without significant disturbance as previously described [25], [42]. Other animals (*n = *2) were not fully acclimated to the aquaria (non-acclimated; N-Ac) and were used for coelomocyte collection after only two weeks in the aquaria. These animals were presumed to be immune activated from contact with open ocean seawater, and the handling associated with collection and shipping.

### *In vivo* Immunological Challenge

A subset of Iq sea urchins (*n = *3) were immunologically activated by two separate injections of LPS (1.0 µg/µl; Sigma-Aldrich Co. St. Louis MO) in artificial coelomic fluid (aCF; see [24]) such that each animal received ∼1 µg LPS per ml of coelomic fluid [43]. The initial injection at 0 hr was followed by a second injection 24 hr later. Samples were collected 24 hr after the second injection. Before immune challenge, animals were Iq, and after injections they were redefined as challenged (Ch).

### Coelomocyte Collection

Whole coelomic fluid (wCF), composed of fluid plus all types of coelomocytes, was withdrawn from the coelomic cavity using a 23 gauge needle and a 1 ml syringe that was pre-loaded with sterile ice cold calcium- magnesium-free sea water (CMFSW-EI; as described [25]), with the addition of penicillin G sodium salt (200 U/ml), streptomycin sulfate (200 µg/ml) and ampicillin sodium salt (25 µg/ml, pH 7.4; antibiotics were obtained from Sigma-Aldrich) for a final dilution of 1∶3 wCF to antibiotic-anticoagulant. The needle was inserted through the peristomium into the coelomic cavity and wCF was withdrawn into the CMFSW-EI solution, expelled into a 1.5 ml tube on ice, and coelomocytes were counted on a hemocytometer. Under sterile conditions, 1.0×10^5^ coelomocytes were either settled onto a circular glass microscope coverslip (18 mm diameter; Fisher Scientific, San Jose, CA; pre-sterilized in 100% ethanol) that had been placed on the bottom of a culture plate well, or were settled directly onto the culture well (12-well polystyrene plate, Corning Inc., Corning, NY). Upon settling and incubation at 14°C for one hr, the phagocytes adhered to the coverslip or the culture well. The CMFSW-EI solution was removed including the non-adherent coelomocytes, and one of several sterile culture media was added to each well (Table 1). The adherent phagocytes were maintained at 14°C for various incubation times.

**Table 1:** Coelomocyte culture media evaluated.

Medium^1^	Components	Comments
aCF	10 mM CaCl_2_, 14 mM KCl, 50 mM MgCl_2_, 398 mM NaCl, 1.7 mM NaHCO_3_, 25 mM Na_2_SO_4_ [24], [38]	***
MB (100%)	3.44% marine broth (Difco), 0.3% yeast extract (Difco) in distilled water [52]	>950 mOsm
MB (5%)	5% MB in distilled water	>950 mOsm
MEM (100%)	MEM* in aCF	>950 mOsm
MEM (5%)	5% MEM in distilled water	>950 mOsm
MB and MEM	2.5% MB and 2.5% MEM in distilled water	>950 mOsm
CCM	0.5 M NaCl, 5 mM MgCl_2,_ 1 mM EGTA, 20 mM HEPES [22]	***
ECCM	0.6% Leibovitz’s L-15 Medium*, 0.87% MEM*, 0.26% F-12 Nutrient Mixture*, 15 mM HEPES, 14.8 mMNaHCO_3_, 3% heat inactivated fetal bovine serum, 10 µg/ml insulin from bovine pancreas, 20 µg/mlcatalase from bovine liver, 55 µM 2-mercaptoethanol, 200 µM L-glutamine, **10% cell free CF**and antibiotics** in aCF	Employed for phagocyte evaluations described here

1 aCF, artificial coelomic fluid; MB, marine broth; MEM, Dulbecco’s Modified Eagle Medium; CCM, coelomocyte culture medium, CF, coelomic fluid, ECCM, echinoid coelomocyte culture medium.

* per suppliers instructions; Gibco™, Invitrogen, Carlsbad, CA.

** 200 U/ml Penicillin G sodium salt, 200 µg/ml Streptomycin sulfate and 25 µg/ml Ampicillin sodium salt.

*** Coelomocytes detached from culture well surface, formed small aggregates and floated in medium after ON (16–21 hr) incubation.

Bolded text indicates components not present in the original cell culture recipe. Other modifications to the original recipe included the omission transferrin, selenous acid and chemically defined lipids (CDL), as well as growth factors, including epidermal growth factor (EGF) and basic fibroblast growth factor (b-FGF).

### Coelomocyte Viability

Cultured phagocytes that had spread on round glass coverslips were dual stained with propidium iodide (PI; 2 µg/ml; Sigma-Aldrich) and live nuclear stain SYTO® 13 (Molecular Probes™, Invitrogen, Eugene, OR) to assess cell viability, following the manufacturer’s instructions. Phagocytes in ECCM from Iq animals were observed after 1 day (*n = *11), 2 days (*n = *11), 3 days (*n = *10), 4 days (*n = *5), 5 days (*n = *5) and 6 days (*n = *5) with an Axioplan fluorescence microscope (Zeiss, Oberkochen, Germany) using 40X NA 0.75 planapochromatic phase contrast objective lens. The Olympus MicroSuite™ B3SV software program was used to quantitate the number of dead cells (PI positive) vs. the total number of cells counted.

### Evaluation of Phagocytes in Culture

#### Morphology

The morphology of unstained phagocytes cultured in various media (Table 1) was evaluated daily over three days on a TMS-F inverted microscope (Nikon Instruments Inc., Melville, NY) using the 40X objective lens. Cells were recorded as settled or otherwise attached to the culture well, and it was noted whether they remained as adherent individual cells or whether they aggregated into suspended three-dimensional spheres. Cultures that contained floating cells after 1 to 3 days were deemed inferior (Table 1) and not evaluated further.

#### Immunological challenge *in vitro*

Settled and adherent phagocytes in ECCM from Iq, Ch or N-Ac animals were incubated in different concentrations of LPS for various periods; 5 min, 15 min, 30 min, 1 hr, 2 hr, 3 hr, overnight (ON; 16–21 hr), 24 hr, 48 hr, and 50–60 hr (50^+^ hr). LPS was added to cultures at different concentrations; 0, 10, 50, or 100 µg/ml of ECCM.

#### Immunocytology

Phagocytes were fixed and stained according to [24]. Briefly, after fixing, washing and blocking, cells were incubated with an equal mixture of rabbit polyclonal anti-185 antibodies (anti-185-66, -68 and -71; 1∶4000 dilution; see [24]) plus mouse monoclonal anti-actin antibody (1∶600 dilution; MP Biomedicals, Solon OH). Cells were post-incubated with goat anti-rabbit-immunoglobulins (GαR-Ig) conjugated to AlexaFluor 568 (1∶400 dilution; Pierce Biotechnology, Rockford IL) mixed with donkey anti-mouse-Ig (DαM-Ig) conjugated to AlexaFluor 488 (1∶200 dilution; Pierce Biotechnology). Cells were mounted with ProLong® Gold Antifade with 4′,6-diamidino-2-phenylindole (DAPI; Invitrogen, Carlsbad CA). Phagocytes were examined with an Axioplan fluorescence microscope (Carl Zeiss, Oberkochen, Germany) and imaged using either a 10X NA 0.25 plan phase, 20X NA 0.5 or 40X NA 0.75 planapochomatic phase contrast objective lens connected to a CCD camera (Hitachi Ltd, Tokyo Japan).

### Statistical Analysis

Cell viability was based on the number of dead cells vs. the total number of cells per sample that were counted on 5–10 different microscope fields. The timing of phagocyte aggregation into syncytia-like structures was evaluated by comparing the numbers of nuclei that were incorporated into syncytia-like structures to the number of phagocytes that were not incorporated, for 5–25 microscope fields per sample. To evaluate the association of Sp185/333 proteins with nuclei for a given sample, the proportion of nuclei associated with Sp185/333 proteins were compared to the total number of cells or nuclei in a field, with 5–15 fields counted. We also determined the proportion of nuclei associated with Sp185/333 proteins that were present within syncytia-like structures compared to cells that were not incorporated into the structures, using 5–25 fields per sample. All statistics were performed using Statistical Analysis Software, SAS, Version 9.1.3 (SAS Inc., Carey, NC). Logarithmic transformation was performed on data sets that displayed a non-normal distribution. Statistical significance was assessed by analysis of variance in general linear models regression procedures (GLM). Bonferroni correction was employed in the pairwise means comparison in order to compare groups of values, including data from different incubation times. Experimental error was addressed by including replicate measurements among model parameters.

## Results

### Echinoid Coelomocyte Culture Medium

**Figure 1:**
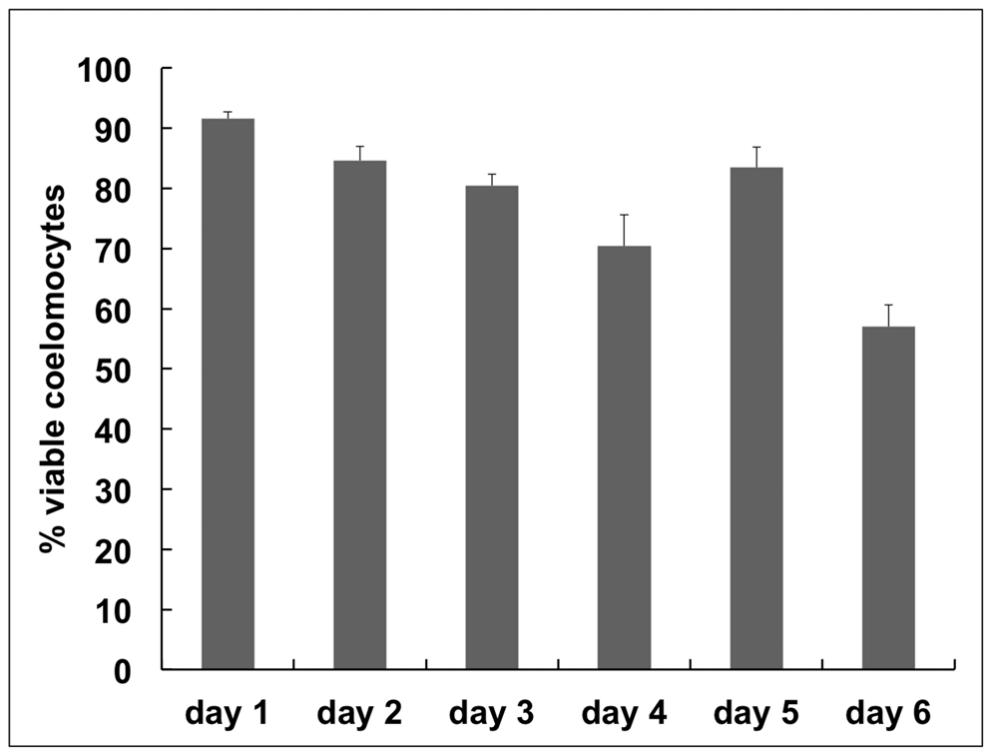
Viability of phagocytes from the purple sea urchin in short-term cultures. Viability decreases from 91.6 to 57.0% over six days. Standard error of the mean (SEM) are shown.

Seven different culture media were tested on phagocytes to determine which one was optimal for subsequent studies of cells in short-term primary cultures (Table 1). Media were selected to match the normal chemical characteristics of coelomic fluid in sea urchins, including a pH close to 7.4 and salinity within the range of 925–950 mOsm at 14°C. Most of the media (5 of 7), including MEM and marine broth, had elevated salinity relative to normal conditions for coelomocytes *in vivo* and were not evaluated with cells in culture. The suitability of the remaining media was evaluated based on the morphology of the cells. Cultures in which phagocytes had settled and spread on the cover slip after ON incubation at 14°C were considered to be healthy, and indicated that the medium could support viable cells. When cells were not spread or attached to the bottom of the culture well after ON culture or for 3 days, the medium was considered sub-optimal (Table 1). The optimal medium in which phagocytes remained well spread for up to three days was echinoid coelomocyte culture medium (ECCM; Table 1) and was employed for subsequent evaluations of phagocytes in culture. ECCM is based on a medium that has been used successfully to culture cells from Polian vesicles and axial organs of *S. droebachiensis* [33]. It was made up of a basal nutrient medium (LDF) containing Leibovitz’s L-15 Medium, Dulbecco’s Modified Eagle Medium and F-12 Nutrient Mixture. ECCM included the original LDF recipe diluted into aCF with modifications, with the addition of 10% cell-free coelomic fluid (cfCF) and the omission of growth factors (Table 1). Viability of phagocytes in ECCM was evaluated every 24 hr for the first 6 days of culture and found to decrease from 91.6% to 57.0% (±5.0%; standard error of the mean; SEM) (Fig. 1).

### Cultured Phagocyte Morphology and Aggregation; Expression of Sp185/333

**Figure 2:**
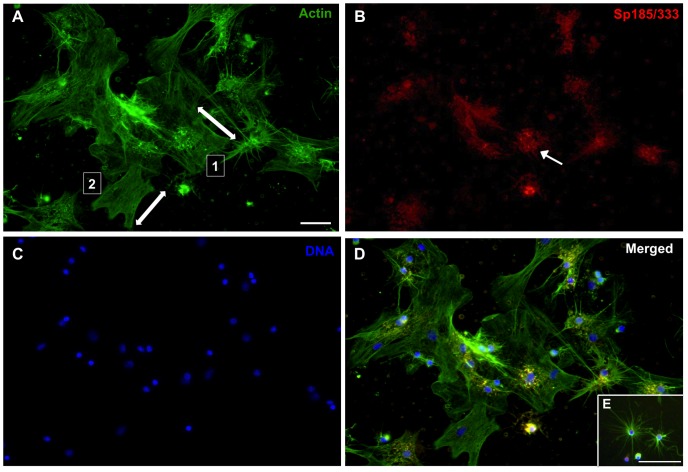
Clusters of phagocytes are present in cultures after 1 hr. Phagocytes are labeled for actin (green, A), Sp185/333 (red, B) and DNA (blue, C). Merged images are shown in D and E. Arrows in A indicate the direction of the actin cables for polygonal phagocytes labeled 1 and 2. The large cell with Sp185/333^+^ perinuclear vesicles (indicated by the arrow in B) is most likely a polygonal phagocyte. Small phagocytes that are not associated with clusters are shown in E. Scale bar is 10 µm.

**Figure 3:**
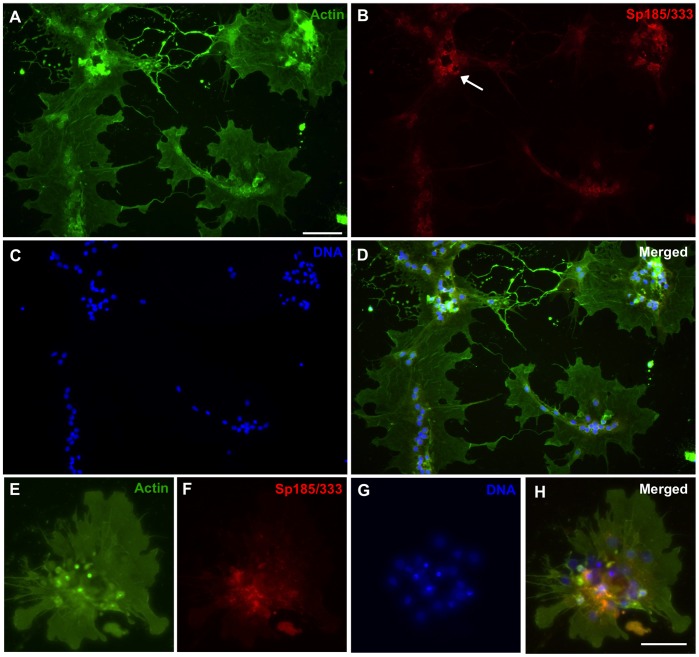
Phagocytes aggregate into syncytia-like structures after 3 hr of incubation. Settled phagocytes were incubated for 3 hr in ECCM, fixed and labeled for actin (green, A, E), Sp185/333 (red, B, F) and DNA (blue, C, G). Merged images are shown in D and H. The morphology of different types of phagocytes is no longer recognizable (compare to Figure 2). Sp185/333 proteins (arrow in B, F) within syncytia-like structures are presumably in perinuclear vesicles, as described previously [24]. Scale bars are 10 µm.

**Figure 4:**
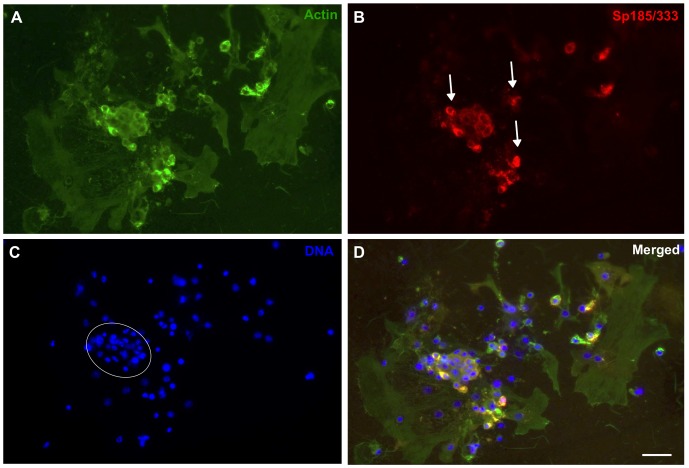
Syncytia-like structures are present after 5 hr of incubation. Settled phagocytes were processed for immunocytology and stained and labeled as in figure 3. Structures contain clustered nuclei associated with Sp185/333 proteins. Arrows in B indicate Sp185/333 proteins (red) in syncytia-like structures. In C, a group of tightly packed nuclei is circled, which are associated with Sp185/333 proteins (D, merged). Scale bar is 10 µm.

**Figure 5:**
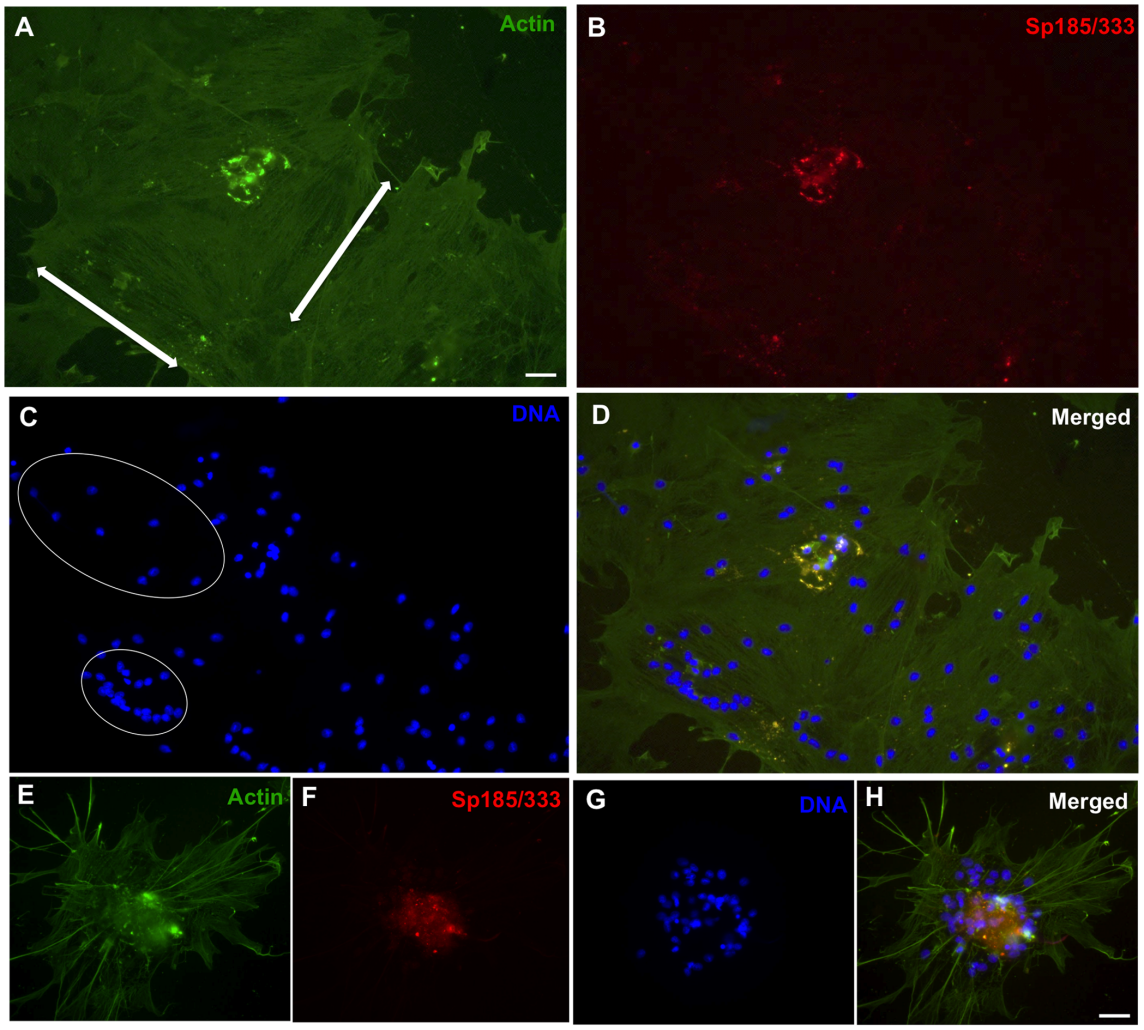
A syncytium-like structure contains both tightly packed and more evenly dispersed nuclei at 24 hr. Settled phagocytes were processed for immunocytology and stained as in Figure 3. Merged images are shown in D and H. Arrows in A mark the direction of actin cables that cross the structure. Sp185/333 proteins (red) associated with syncytia-like structures are present in the center of B. Tightly packed nuclei (smaller circle in C) and more evenly dispersed nuclei (larger circle in C) are both present within a syncytium-like structure. Sp185/333 proteins are associated with the clustered nuclei in a syncytium-like structure (E–H). Scale bars are 10 µm.

Coelomocytes from both the purple and the green sea urchin (*Strongylocentrotus droebachiensis*) in addition to hemocytes from marine decapods aggregate in culture after several hours and are assumed to form syncytia or multinucleate giant cells lacking cellular boundaries between neighboring nuclei [5], [8], [9], [11], [13], yet there is no explicit experimental evidence to support cell fusion. Phagocytes in short-term cultures from *S. purpuratus* form small aggregates by 1 hr within which small phagocytes and polygonal cells expressing Sp185/333 proteins are identified [24]. Some aggregates appear to be composed almost entirely of Sp185/333^+^ phagocytes. Nonetheless, the morphology of Sp185/333^+^ cells in culture has not been examined for periods longer than 2 hr, nor have quantitative data been collected on the aggregation of cultured Sp185/333^+^ phagocytes [24], [34]. To address the question of whether the immune Sp185/333 proteins play a role in aggregation, we began by examining phagocytes in culture for periods longer than 1 hr, and evaluated the presence of Sp185/333 proteins and cell morphology by immunocytology. At 1 hr, small groups of phagocytes included polygonal cells (Fig. 2A–D) and small phagocytes (Fig. 2E), of which some were Sp185/333^+^. The cytoskeletal organization of individual polygonal cells showed actin cables oriented in different directions (Fig. 2A, arrows) suggesting that they were individual cells, and small phagocytes displayed their usual filopodial morphology (Fig. 2E) (see [18], [24]). After 3 hr of incubation, the phagocytes had aggregated into syncytia-like structures in which the nuclei were non-randomly distributed and tightly clustered (Fig. 3). This was consistent with putative fusion of the plasma membranes and the movement of nuclei to central regions of the syncytia. Some syncytia-like structures contained Sp185/333 proteins that were co-localized with clustered nuclei (Fig. 3B, D, F, H). At 5 hr, many nuclei remained tightly clustered within syncytia-like structures (circled in Fig. 4C), and the Sp185/333 proteins remained co-localized with some of the clustered nuclei (Fig. 4B, D). At 24 hr, the nuclei in the syncytia-like structures where either tightly clustered or more dispersed (Fig. 5), and the Sp185/333 proteins co-localized with the clustered nuclei in some of the structures (Fig. 5E–H). This nuclear movement in the syncytia-like structures is in agreement with observations of cultured phagocytes from the green sea urchin [8]. Within a single syncytium-like structure the actin cables were oriented across the entire structure in one direction, wherein the nuclei were either tightly clustered or more evenly dispersed. However, some syncytia-like structures had actin cables oriented in several directions, suggesting that the cytoskeletons of individual syncytia had subsequently fused to one another after their initial formation (Fig. 5A, arrows). We interpret these results to indicate that phagocytes may have formed syncytia in culture, beginning at about 3 hr. Although Sp185/333^+^ small phagocytes and polygonal cells could not be discerned, Sp185/333 proteins within syncytia-like structures were identifiable at all incubation times and appeared to be associated with some of the clustered nuclei in the syncytia-like structures (Fig. 5C, 5H). Overall, these results were similar to the timing of syncytia formation of coelomocytes reported for the green sea urchin in which the cells aggregated into syncytia as early as 4 hr in culture, and by 6 hr, the cell boundaries within the syncytia were not identifiable [9]. Results shown here suggested that by 3 hr the plasma membranes of neighboring phagocytes may fuse during cell aggregation to form syncytia in short-term cultures.

### The Rate of Aggregation is Accelerated by LPS

**Figure 6:**
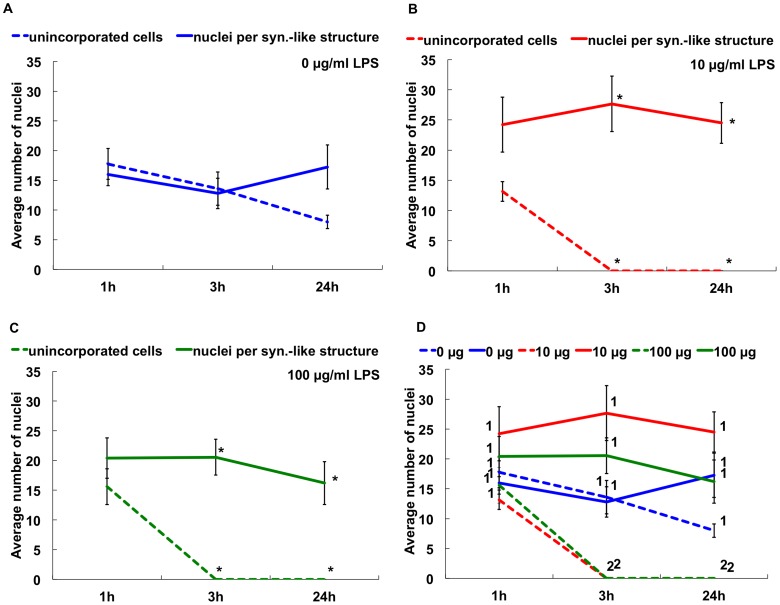
Phagocyte aggregation rate increases with exposure to LPS in culture. Settled phagocytes from Iq animal #2 were incubated with 0 (A), 10 µg (B), or 100 µg (C) LPS/ml ECCM and the number of cells within aggregates was compared to non-aggregated cells. D. All results are shown together for direct comparisons. The dashed lines represent cells that are not incorporated into syncytia-like structures and the solid lines represent the number of nuclei incorporated per syncytium-like structure. There are significantly more aggregated phagocytes than non-aggregated phagocytes after 3 hr and 24 hr of exposure to 10 (B) or 100 (C) µg LPS/ml compared to shorter incubation times (asterisks; *P<*0.05). Lines marked in D with the same number (1 or 2) are not significantly different. SEM are shown.

Although the mechanism driving cellular aggregation is not known, phagocytes in culture aggregate in response to contact with foreign materials such as glass, plastic or bacteria, and this may be an aspect of the encapsulation response [8], [11]. While the timing of invertebrate immune cell aggregation in the presence of bacteria has been described [9], [10], [15], it has not been quantified. We therefore evaluated the timing of aggregation quantitatively with respect to the addition of LPS in the cultures of phagocytes from Iq animal #2 (Table 2). Aggregation was examined at 1, 3 and 24 hr with and without LPS. Phagocytes aggregated more quickly when exposed to LPS (*P<*0.05; Fig. 6), and in the presence of LPS all phagocytes were incorporated into syncytia-like structures by 3 hr and remained aggregated at 24 hr. Thus phagocytes respond to LPS in the absence of proteins from the coelomic fluid and show an increased rate of aggregation.

**Table 2:** Coelomocytes cultured with different concentrations of LPS for different times.

Animal	Injection	Collection Time	LPS concentration (µg/ml)	LPS incubation time in culture
Iq 1	−	−	0, 10, 100	5 m, 15 m, 1 h
			0, 50, 100	15 m
			0, 10, 50, 100	30 m
Iq 2	−	−	0, 10, 100	1 h, 3 h, 24 h
Iq 3	−	−	0	24 h
N-Ac 4	−	−	0, 10, 50, 100	30 m, 1 h, ON
N-Ac 5	−	−	0, 10, 50, 100	30 m, 1 h
			0, 10, 100	ON
Ch 6	LPS	post	0, 10, 50, 100	ON, 48 h, 48^+^h
			0, 50, 100	24 h
Ch 7	LPS	pre	0, 10, 50, 100	30 m, 1 h, ON, 24 h, 48 h
		post	0, 10, 50, 100	30 m
			0, 10, 50, 100	1 h, 2 h, 3 h, ON, 48 h
			0, 10, 50	24 h
Ch 8	LPS	pre	0, 10, 50, 100	30 m, 1 h, 24 h, 48 h
			0, 10, 50	ON
		post	10, 100	30 m
			0, 10,50, 100	1 h, 2 h, ON, 48 h
			0, 10, 100	3 h
			0, 10, 50	24 h

Coelomocytes were settled in culture wells for 1 hr prior to experimental treatments (i.e., with or without LPS).

LPS, lipopolysaccharide; Iq, Immunoquiescent; N-Ac, Non-Acclimated; Ch, Immune challenged; ON, 16–21 hr; post, some animals were injected with LPS prior to collection of coelomocytes for cultures; pre, coelomocytes were collected from some animals prior to injection with LPS.

### The Proportion of Sp185/333 Proteins Associated with Nuclei Increases after LPS Exposure

**Figure 7:**
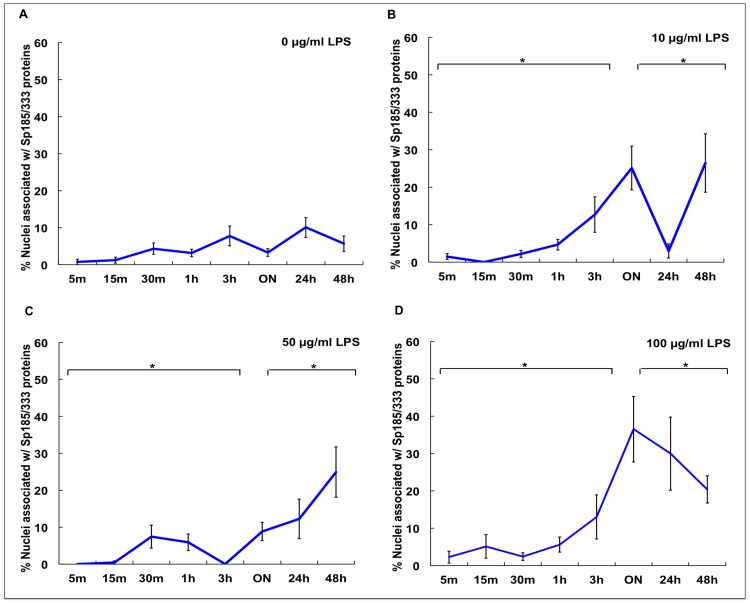
The proportion of nuclei associated with Sp185/333 proteins in cultured phagocytes increases after exposure to LPS. Phagocytes from Iq animals (*n* = 4) were incubated with 0 µg (A), 10 µg (B), 50 µg (C), or 100 µg (D) LPS/ml in ECCM over time. The percentage of nuclei associated with Sp185/333 proteins is significantly higher after incubation with 100 µg LPS/ml (D) compared to 0 (A), 10 (B) or 50 (C) µg LPS/ml (*P<*0.05). There is a significant increase in the percentages of nuclei associated with Sp185/333 proteins when cultures are incubated with LPS (all concentrations) for ON to 48 hr compared to cultures incubated for shorter periods of up to 3 hr (brackets with asterisks; *P<*0.05). ON indicates 16–21 hr. SEM are shown.

**Figure 8:**
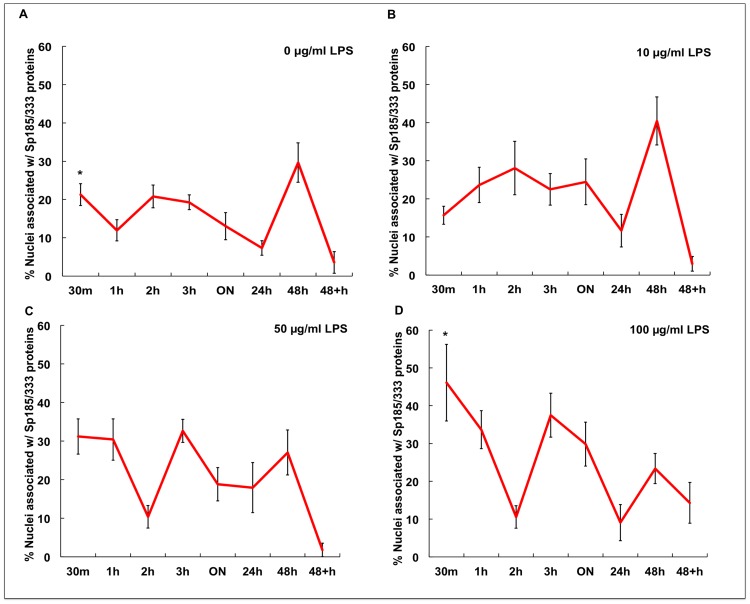
The proportion of nuclei associated with Sp185/333 proteins in cultured phagocytes from Ch animals increases after *in vitro* exposure to LPS. Phagocytes from Ch animals (*n* = 3) were incubated with 0 (A), 10 (B), 50 (C) or 100 (D) µg LPS/ml ECCM over time. There is a significant increase in the percentage of nuclei associated with Sp185/333 proteins when exposed to 100 µg/ml LPS (D) compared to 0 µg/ml LPS (A; *P<*0.05; asterisks) regardless of incubation time. SEM are shown.

**Figure 9:**
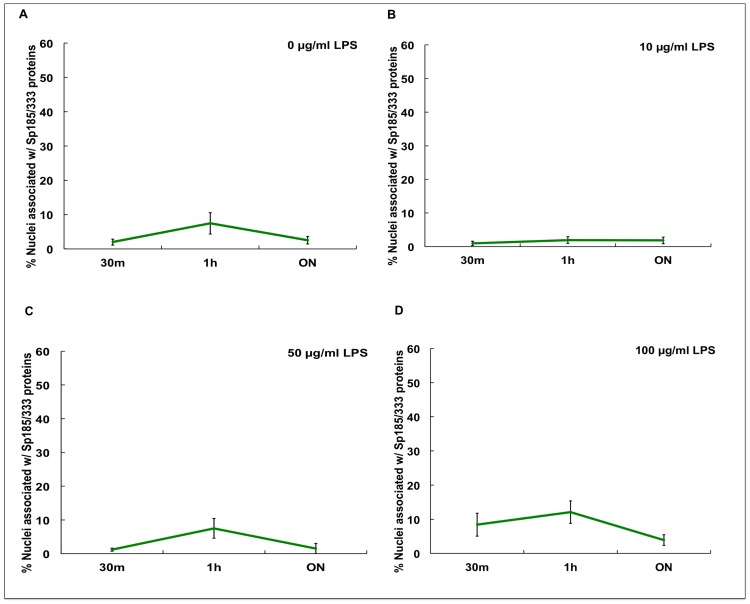
The proportion of nuclei associated with Sp185/333 proteins in cultured phagocytes from N-Ac animals does not change after exposure to LPS *in vitro*. Phagocytes from N-Ac animals (*n* = 2) were incubated with 0 µg LPS/ml (A), 10 µg LPS/ml (B), 50 µg LPS/ml (C), or 100 µg LPS/ml (D) in ECCM. There are no significant differences among cultures treated with or without LPS. SEM are shown.

**Figure 10:**
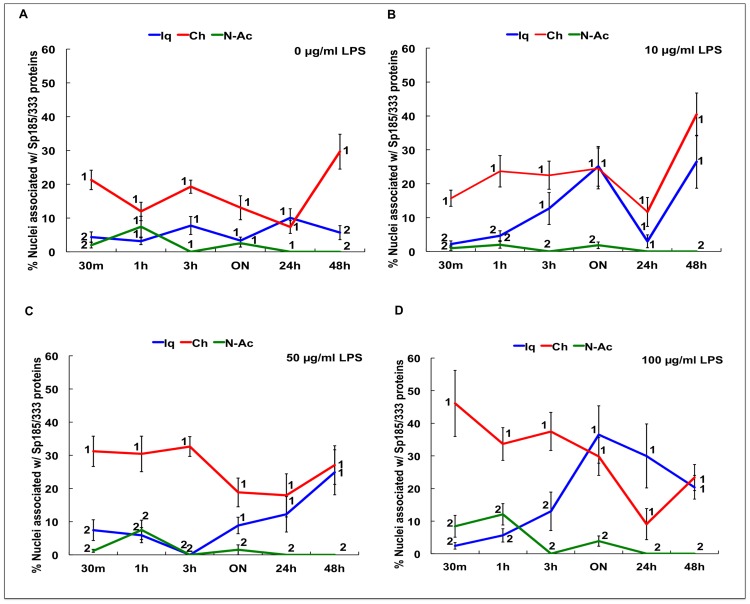
The proportion of nuclei associated with Sp185/333 proteins in cultured phagocytes from Iq, Ch and N-Ac animals is variable when coelomocytes are exposed to LPS. The data in Figures 7–9 are presented together for direct comparisons. Phagocytes were exposed to 0 (A), 10 (B), 50 (C) or 100 µg LPS/ml (D). Significant differences are indicated by numbers (1 or 2; *P<*0.05) associated with the data points. Lines marked with the same number are not significantly different. SEM are shown.

The proportion of Sp185/333^+^ cells is known to increase significantly *in vivo* 24 hr after immune challenge [24], [41]. However, it is not known whether phagocytes are capable of generating a measurable increase in Sp185/333 proteins in culture in response to LPS, resulting in either an increase in the number of Sp185/333^+^ phagocytes and/or an increase in the amount of Sp185/333 proteins in cells. To address these possibilities, cells from Iq, Ch and N-Ac animals were incubated with different concentrations of LPS for various times (Table 2), and Sp185/333^+^ cells were enumerated by immunocytology. Because different coelomocyte types could not be discerned in culture at 3 hr and later (see above), nuclei associated with the Sp185/333 proteins within syncytia-like structures were counted instead of Sp185/333^+^ cells. In general, results indicated that the percentages of nuclei associated with the Sp185/333 proteins in cultured phagocytes increased in the presence of LPS (Figs. 7–10). In the absence of LPS, there was no marked difference in the proportion of nuclei associated with Sp185/333 proteins at any time point in cultures from Iq animals (Fig. 7A). However, for cultures of phagocytes from Iq animals incubated with 100 µg/ml LPS, there was a significantly greater proportion of nuclei associated with the Sp185/333 proteins at all incubation times compared to cultures with lower concentrations of LPS (*P<*0.05; Fig. 7B–D). In general, syncytia-like structures in older cultures showed more clustered nuclei associated with Sp185/333 proteins compared to cultures at earlier time points (Fig. 7; *P<*0.05). When phagocytes were collected from Ch animals, which had been injected with LPS prior to being placed in culture, there was a greater proportion of nuclei associated with Sp185/333 proteins in cultures exposed to 100 µg LPS/ml compared to control cultures without the addition of LPS (Fig. 8). Cultured phagocytes from the N-Ac animals showed less association of the Sp185/333 proteins with nuclei. Furthermore, the level of exposure to LPS or no exposure to LPS did not change the proportions of nuclei associated with Sp185/333 proteins (Fig. 9). Thus, phagocytes from Iq and Ch animals were capable of responding to LPS in culture, although this was not observed for cells from N-Ac animals.

The proportions of nuclei with associated Sp185/333 proteins for phagocytes cultured in the presence or absence of LPS (Figs. 7–9) were combined for comparison to identify similarities and differences among the Iq, Ch and N-Ac animals (Fig. 10). Phagocytes from Ch animals had a greater proportion of nuclei associated with Sp185/333 proteins at earlier time points and continued to express the proteins until the cultures were terminated. In contrast, phagocytes from Iq animals, which were not immune challenged prior to collection, responded to LPS after ON exposure. Relative to cells from both Ch and Iq animals, phagocytes from N-Ac animals generally had fewer nuclei associated with Sp185/333 proteins when cultures were compared at the same time points. This suggested that the N-Ac animals, which had not been immune challenged prior to coelomocyte collection, were not as immunologically activated as the Ch animals. From these results, we infer that phagocytes responded to LPS in culture, that phagocytes from Iq animals responded after ON incubation, while those from Ch animals had responded to LPS *in vivo* and continued to express Sp185/333 proteins in culture. This resulted in phagocytes from Ch animals with an increased proportion of nuclei associated with Sp185/333 proteins at the time of the first measurement (30 min) and was maintained for 48 hr in culture.

### Sp185/333 Proteins are not a Major Driving Force for Phagocyte Aggregation

**Figure 11:**
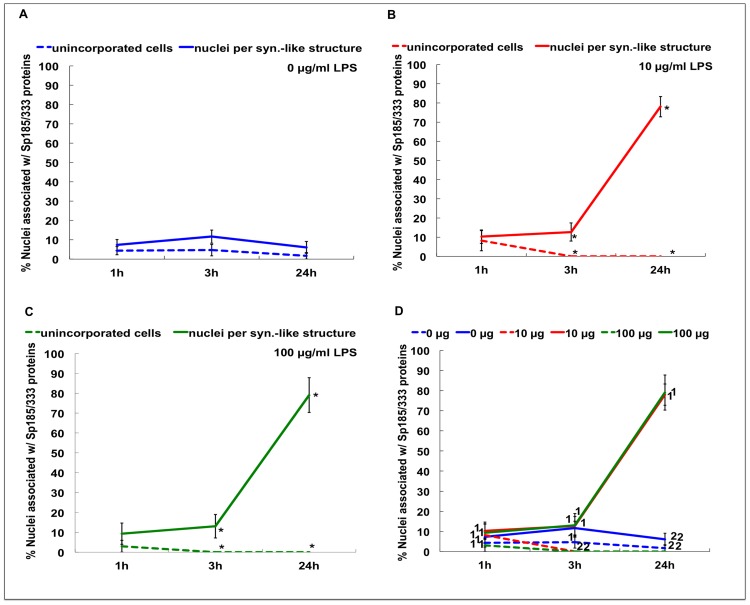
The percentage of nuclei associated with Sp185/333 proteins increases when phagocytes are exposed to LPS in culture. Coelomocytes from Iq animal #2 were settled for 1 hr onto culture well plates and incubated with 0 µg (A) 10 µg (B) or 100 µg (C) LPS/ml ECCM for different times. D shows combined data from A, B and C. There is a significant increase in the proportion of nuclei associated with Sp185/333 proteins within syncytia-like structures vs. non-aggregated Sp185/333^+^ phagocytes after 3 h and 24 hr exposure to 10 (B) or 100 (C) µg LPS/ml, compared to results for 1 hr (asterisks; *P<*0.05). Significant differences are shown in D where data points are marked with a different number (1 or 2). Lines in D with the same number are not significantly different. SEM are shown.

We have shown here that both phagocyte aggregation and the proportion of nuclei associated with Sp185/333 proteins increased when cultures included LPS. Previously, it had been shown that purple sea urchin phagocytes aggregate in less than a day in CCM (Table 1) [18], [24]. Although syncytia-like structures include Sp185/333 proteins [24], the timing of aggregation and the correlation with the presence of Sp185/333 proteins was not examined. To determine whether the Sp185/333 proteins were involved in aggregation, we asked whether exposure to LPS would alter the proportion of nuclei associated with Sp185/333 proteins within syncytia-like structures compared to the proportion of unincorporated phagocytes that were Sp185/333^+^. Phagocytes from Iq animal #2 were cultured in the presence or absence of LPS for 1 to 24 hr (Table 2). In the absence of LPS, there were no differences between the proportions of nuclei associated with Sp185/333 proteins within syncytia-like structures compared to unincorporated Sp185/333^+^ cells for all time points (Fig. 11A). After exposure to LPS for 1 hr, there was no difference between the total number of unincorporated phagocytes relative to those incorporated into syncytia-like structures and 90% of the phagocytes were Sp185/333^–^ (Fig. 11B–D). However, by 3 and 24 hr of LPS exposure, all the phagocytes had become incorporated into syncytia-like structures, with no unincorporated phagocytes remaining in the culture. Although all of the phagocytes were in syncytia-like structures by 3 hr, only ∼10% of the nuclei had associated Sp185/333 proteins. In contrast, after 24 hr of incubation with LPS, ∼90% of nuclei within syncytia-like structures were associated with Sp185/333 proteins (*P<*0.05; Fig. 11B–D). These results indicated that phagocytes from an Iq animal with low expression of Sp185/333 proteins formed syncytia-like structures, and that they formed faster in response to LPS. However, there was no correlation between the initiation of syncytia-like structure formation and Sp195/333 expression during the early time points of culture. Furthermore, by 24 hr of incubation with LPS there was an increase in the expression of Sp185/333 and a significant increase in the percentage of nuclei that were associated with Sp185/333 proteins.

## Discussion

A new method for short-term culture of coelomocytes from the purple sea urchin, *S. purpuratus*, has enabled us to evaluate phagocyte behavior and activities *in vitro*. Cultured phagocytes aggregate into syncytia-like structures that are discernible by 3 hr and persist until the culture is terminated. Exposure to LPS for 3 hr accelerates phagocyte incorporation into syncytia-like structures, occurring significantly faster than in the absence of LPS. LPS also significantly increases the number of phagocyte nuclei that are associated with Sp185/333 proteins after 24 hr. This suggests that although the Sp185/333 proteins may not function in phagocyte-phagocyte interactions that lead to aggregation, expression of these proteins by phagocytes appears to be induced by LPS. Their association with clustered nuclei has not been observed previously and the functional significance of this observation is not known.

### Phagocytes Likely form Syncytia in Culture

Syncytia formation has been reported for various marine invertebrate immune cells in culture including echinoderm phagocytes incubated on flat surfaces as well as in suspended hanging drops [5], [8], [9], [11], [13]. On flat surfaces, cells have been reported to aggregate into syncytia, beginning with plasma membrane fusion between neighboring cells [13]. This process appears to continue until many cells have been incorporated into a syncytium resulting in giant multinucleated structures [5], [8], [9], [11], [13]. Large multinucleated syncytia have been repeatedly observed in culture. The continuity of the cytoskeletons within these structures is taken to imply that cultured phagocytes from sea urchins undergo plasma membrane fusion between neighboring cells.

Phagocyte aggregation among *S. purpuratus* cells is similar to that reported for *S. droebachiensis* in which the positioning of nuclei within and among syncytia-like structures in cultures of up to 24 hr range from tightly clustered to more evenly spaced [9]. Bertheussen and Seljelid (1978) and Bertheussen [8] reported similar events: coelomocyte cultures older than 24 hr form syncytia with either regularly dispersed nuclei or more closely clustered nuclei. The presence of tightly clustered nuclei is strong evidence that the plasma membranes of adjacent cells fuse, which enables the nuclei to move to the center of the fused cytoplasm. In *S. purpuratus,* the syncytia-like structures containing either tightly clustered or more evenly dispersed nuclei (at 5 hr), or both (at 24 hr), show cytoskeletal morphology with actin cables oriented in uniform direction(s) across an entire multinucleated structure. This is further support that plasma membrane fusion occurs between adjacent cells resulting in genuine syncytia. Fusion may also occur between neighboring syncytia to form progressively larger multinucleated structures that show actin cables oriented in multiple directions. Because we observe some nuclei that are clustered in syncytia-like structures and other nuclei that are evenly spaced, we speculate that they may be continuously rearranging into and out of tight clusters over time.

### Coelomocyte Aggregation in Culture is Likely an Immune Response

The signal that induces cultured invertebrate immune cells to form syncytia-like structures is not known. It is thought that cells detect the foreign surface on which they are cultured and form progressively larger multinucleated structures in a coordinated effort to encapsulate or wall off the entire foreign surface, i.e., the glass or plastic surface [9], [44], [45]. This is likely the same basic encapsulation response of phagocytes that occurs *in vivo* in which cells coordinate to cover a wound, to wall off an infected area, or to encapsulate agglutinated microbes or a parasite. This is observed *in vivo* when a mass of coelomocytes surrounds a piece of glass [8], [9] or plastic [13] that is experimentally inserted into the body of an adult echinoid. The encapsulating phagocytes appear to form syncytia around the foreign material. The first documented evidence of this basic, primary innate immune response stems back to Elie Metchnikoff and his experiments on bipinnaria larvae of a sea star. He demonstrated that amoeboid-like phagocytes encapsulate and phagocytose rose pickles or glass rods inserted into the larval blastocoel [46].

Encapsulation in adult echinoderms is assumed to be initiated by coelomocyte detection of a pathogen or parasite and is initiated by cellular clot formation. Cellular clots are induced upon collection of coelomic fluid in the absence of anticoagulant [11], [29], however the molecular basis for cell clotting in sea urchins is not well understood. There are a number of gene models in the genome of the purple sea urchin encoding proteins that are likely involved in cell-cell adhesion [47] and cell clotting or coagulation of the coelomic fluid including amassin, thrombin, plasminogen, serpins, kallikrien and transglutaminase [4], [48]. Amassin is expressed by immune activated coelomocytes [37] and initiates intercellular clot formation through disulfide bond formation [30]. Clusters of coelomocytes settled on glass have amassin localized between neighboring cells. Consistent with the observations of Hillier and Vacquier [30], when phagocytes have been kept in culture for 1 hr, they begin to form small aggregates that may be mediated by amassin that is produced by the cells. Arylsulfatase, which is also expressed by coelomocyes [49], has dual functions in both lysosomes and on phagocyte surfaces and is involved in cell adhesion and clot formation of activated, filipodial phagocytes [31], [32]. Amassin and arylsulfatase, which function in cell-cell adhesion of coelomocytes, likely work in parallel and may both be produced by phagocytes in culture and act to initiate cell interactions that lead to the formation of syncytia-like structures.

Phagocytes cultured with LPS aggregate more quickly compared to cultures without LPS. Coelomocytes from *S. droebachiensis* in hanging drop cultures respond to the addition of Gram-negative bacteria by forming “clumps” over time beginning at 5 min [10]. At 2 hr, the bacteria uniformly spread out from the site of inoculation into the hanging drop, and cover a large area of the culture. In response, the coelomocytes form a wall-like clot around the outer edges of the bacterial mass, apparently walling off and blocking the bacterial spread. Although the structure of the culture containers differs in these two studies (i.e. hanging drop vs. flat surface), results are in accord with and support the notion that phagocytes can detect and respond to bacteria in culture, and that this can occur as quickly as 5 min. Because the effects of LPS and bacteria added to cultures have been evaluated in the absence of proteins from the coelomic fluid, the cellular response suggests the presence of a phagocyte receptor for LPS that induces immune activation. LPS detection may be mediated by one or more Toll-like receptors that are encoded in the sea urchin genome by 253 genes [50]. Detection of bacteria by homologues of peptidoglycan recognition proteins and Gram-negative binding proteins, which are encoded by genes that have been annotated in the genome [48], may also function to induce the phagocyte responses to microbes in culture.

### Role of Sp185/333 in the Formation of Syncytia-like Structures

Sp185/333 proteins have been speculated to play a role in the initial stages of cell-cell interactions and aggregation of cultured phagocytes. This was based on images of aggregations of Sp185/333^+^ small phagocytes held *in vitro* for 1 hr [18], [24]. This notion is supported by the localization of the Sp185/333 proteins on the surface of small phagocytes and the tendency of the Sp185/333 proteins to multimerize [24], [36]. This cell surface localization and multimerization activity has been speculated to initiate cell-cell associations that result in aggregation leading to syncytia-like structures. We addressed this issue using quantitative analysis while expanding the incubation times beyond that of the previous reports [24], [51]. Although aggregations of small phagocytes at very early time points are positive for Sp185/333 proteins, after 3 hr in culture our results suggest that the Sp185/333 proteins are not required for the formation of syncytia-like structures. The majority of the phagocytes in culture do not express Sp185/333 proteins prior to being incorporated into syncytia-like structures, and there is no Sp185/333^+^ cell surface staining on cultured phagocytes after 1 hr. Rather, we observed Sp185/333 proteins associated with clustered nuclei at 3 hr and later. Although our results differ from those of Brockton et al. [24], the cells were imaged at different times (1 hr or less vs. 3 hr and longer). Furthermore, different sea urchins were used in these two studies and these animals are known to have different levels of Sp185/333 protein expression, different percentages of Sp185/333^+^ cells, and different levels of immune activation that is associated with different rates of aggregation of cells in culture (AJM, personal observations). Differences in the culture media employed here and that used by Brockton et al. [24] may also affect the behavior of the cells in culture. Results reported here indicate that the Sp185/333 proteins do not appear to be a driving force for phagocyte aggregation in culture and that these proteins may be involved in other immune responses such as microbial opsonization (CM Lun, C Schrankel, S Sacchi and LC Smith, unpublished; HY Chou and LC Smith, unpublished).

### Conclusions

Cell aggregation *in vitro* likely reflects a normal encapsulation response of the sea urchin immune system. While glass or plastic surfaces alone may induce a basal rate of encapsulation, LPS increases the rate of aggregation. The event of cell aggregation is presumably immune related, but in the absence of a pathogen associated molecular pattern the process takes longer. Although it is not known whether protein(s) other than amassin and/or arylsulfatase may mediate cell aggregation, our results suggest that Sp185/333 proteins are not likely to be involved. However, their association with nuclear clusters in syncytia-like structures is a new and unexpected result. Overall, empirical data indicate that the diverse Sp185/333 proteins are involved with the innate immune system (reviewed in [34]), however their functions remain to be identified.
